# Effects of weaning biscuits on the nutritional profile and the cognitive development in preschool children

**DOI:** 10.1186/1824-7288-36-18

**Published:** 2010-02-18

**Authors:** Peerkhan Nazni, Subramanian Pradheepa, Abul Hasan

**Affiliations:** 1 Department of Food Science, Periyar University, Salem, Tamilnadu, India; 2 Consultant Pediatric Surgeon, City hospital, Erode, Tamilnadu, India

## Abstract

**Aim:**

To study the effect of weaning biscuits supplementation of the nutritional parameters and cognitive performance of the selected children.

**Methods:**

Three primary schools situated in Salem District, Tamilnadu, India were selected. A total number of 150 school children, 61 from primary school I, 46 from primary school II and 43 from primary school III comprised the study sample. About 80 primary school children with Grade II malnutrition were selected for the experimental study. Home diet without any supplementation was followed by Group I (n = 20, control group), potato flour biscuit was supplemented to Group II (n = 20), wheat biscuits was given to Group III (n = 20) and ragi biscuits were given to Group IV (n = 20) for the period of 3 months. Parameters like anthropometric measurements, hemoglobin content clinical picture and cognitive performance were analyzed before and after supplementation.

**Results:**

Results about Group I (control group) showed no significant difference in height, weight and clinical picture and cognitive performance after three months on their home diet. In Group II, III and IV significant increase in all the above parameters was noticed. More increase was found in Group II children supplemented with potato flour biscuits for a period of 3 months. About cognitive performance better results was obtained in Group II followed by Group III (supplemented with wheat biscuits) and Group IV (supplemented with ragi biscuits). Least was obtained by control group children who are in their home diet.

**Conclusion:**

All these observations evident that if such weaning biscuits made with potato flour, wheat and Ragi can form a daily ingredient in their diets, it will bring out better all round development of the children.

## Aims

In India, Protein Energy Malnutrition(PEM) among children under five years still constitutes a major public health problem, poor infant feeding and weaning practices are major contributory factors in malnutrition especially among the under fives. Weaning diets of the low income families are often cereal gruels or regular family diet with or without small quantities of pulses thus inadequate in both calories and proteins. As a result, prevalence of PEM along with other micronutrient deficiencies is the order of the day [1].

Potato (*solanum tuberosum *L) belongs to family solanaceae in one of the major tuber corps of the world and most important commercial vegetable vis-à-vis food crop grown in India. It is rich in carbohydrates and good quality proteins namely tyrosine, proline, leucine and isoleucine responsible for the good nutritional status. It is are available in India round the year and is a good supplement to the cereal diets. Potato based products can be more well balanced in proteins, vitamins and calories/unit area/unit time than any other food [2].

Wheat grains are used to make flour, which is transformed into dough and then breads, which is a staple food in every country and culture. It is rich in protein and provides daily requirement of energy. It has wide culinary uses, from the making of breads, pasta and cakes to fermentation of alcoholic beverages. Finger millet is especially valuable as it contains the amino acid methionine which is lacking in the diets of hundreds of millions of the poor who live on starchy staples such as polished rice or maize meal. In southern parts of India, pediatricians recommend finger millet based food for infants of six months and above because of its high nutritional content especially calcium[3].

Soy being an excellent source of both calories and good quality protein along with other micronutrients can in a large measure help formulation of nutrient dense weaning and supplementary foods that can be recommended for consumption if mitigation of PEM is the concern [4].

The aim of the study is to develop and standardize the weaning biscuits, to study background information, clinical history, nutritional status and dietary pattern, to evaluate the supplementary effect on anthropometric and cognitive development of the selected children.

## Methods

Initially good quality potatoes were taken, washed, peeled, pre treated with (3% Nacl with 0.05% ascorbic acids) sliced, blanched, cooled and once again treated with 0.02% potassium meta bisulphate, drain the treated slices, dry in sun and made into flour. The other ingredients like soya flour, ragi flour, and wheat flour were procured from the local market. Three varieties of weaning biscuits were developed using the proportion of Potato flour (Variety I) Wheat flour (Variety II) Ragi flour (Variety III) with soya flour in the ratio of 80:30 with the addition of sugar and fat. All the developed three varieties of biscuits were evaluated for their acceptability by a panel of 10 judges from Department of Food Science, Periyar University, Salem. All the judges were asked to score the products for appearance, colour, flavor, taste, texture and over all acceptability using a score of a nine point hedonic scale [5]. The developed biscuits were analyzed for its nutritive value with reference to calories, protein, fat, carbohydrates, crude fiber and total ash using the standard procedures [6].

Three primary schools situated in Omalur, Salem district, Tamilnadu, India were selected by purposive sampling method for the sample selection. A total of 150 preschool children 61 from primary school I, 46 from primary school II, 43 from primary school III, belonged to the age group of 2 - 3 were selected for the initial survey. After preliminary survey, 80 preschool children were identified as malnourished (Grade II) based on the height and weight measurement. Assessment of mean nutrient intake using food weighment survey of selected malnourished children was found to be low as energy-710 ± 23.45 K.cal, protein-10 ± 0.92 g and fat-8 ± 1.20 compared to the Recommended Dietary Allowances (RDA) and this calorie gap in their dietaries was estimated as 320 k.cal. These 80 preschool children are admitted for the experimental study.

Identified Eighty Grade II malnourished primary school children were divided into four groups with twenty subjects in each group and given the following weaning biscuits.

Group I: 20 children kept as control group they are on home diet only.

Group II: 20 children supplemented with Potato flour biscuits.

Group III: 20 children supplemented with Wheat biscuits.

Group IV: 20 children supplemented with Ragi biscuits.

Each supplemented groups received six biscuits comprising the weight of 60 grams daily. Three biscuits were given in the mid morning and three biscuits in the evening. This whole research procedure was compatible with Helsinki Declaration and written consent was received from all the participants.

Before starting the feeding trail one local school teacher from each school were selected to distribute and monitor the feeding for the selected children of their area. They were initially briefed about the importance of the study. The feeding trail was conducted for a period of 3 months and during the entire study period, all the experimental and control groups were supervised by the research staff. The study was approved by the Institutional Ethical Committee (IEC) members.

Background details such as age, sex, type of family, family size, birth order, level of income per month were elicited by interviewing the parents of the selected children using an interview schedule.

Anthropometric measurements like height and weight, clinical picture were studied before and after supplementation with the help of a physician. Apart from above data certain perceptual development that measures cognition was assessed before and after the supplementation period for the selected children using specially designed criteria [7].

The data was complied and analyzed by using statistical methods. Descriptive statistics ANOVA and paired comparison test are computed using a statistical software SPSS version 15.0 Duncan's multiple range tests is applied to determine the significant differences between the supplemented biscuits.

## Results

### Organoleptic evaluation of the developed biscuits

The results on organoleptic parameters is given in Table-1

**Table 1:** Organoleptic evaluation of the developed biscuits

Type of Variations	Appearance	Colour	Flavour	Texture	Taste	Overall acceptability
Potato biscuits	7.88 ± 0.78^b^	7.20 ± 0.63^ab^	8.30 ± 0.48^b^	8.40 ± 0.51^b^	8.20 ± 0.78^b^	8.40 ± 0.51^b^

Wheat biscuits	8.00 ± 0.81^b^	7.00 ± 0.81^a^	7.00 ± 0.81^a^	6.90 ± 0.87^a^	6.50 ± 0.52^a^	7.20 ± 0.63^a^

Ragi biscuits	6.80 ± 0.63^a^	7.70 ± 0.67^b^	7.70 ± 0.67^b^	6.90 ± 0.73^a^	7.60 ± 0.84^b^	8.00 ± 0.81^b^

F-ratio	7.342	2.562	9.369	14.261	13.841	8.400

P-Value	0.003**	0.006**	0.001**	0.000**	0.000**	0.001**

** - Significant at 0.01% level

* - Significant at 0.05% level

NS - not significant

Values with different superscripts are significantly different from each other on application of Duncan multiple Range test.

Among the three variation wheat biscuits has got highest score of 8.00 followed by the potato biscuits with the score of 7.88 and the least score 6.80 is obtained by ragi biscuits for appearance. Regarding colour attributes the highest score 7.70 is obtained by ragi biscuits followed by potato biscuits is 7.80 and the least score of 7.00 is obtained by wheat biscuits. For the flavour attributes potato biscuits has got highest score of 8.30 followed by ragi biscuits with the score of 7.70 and the least score 7.00 is obtained by wheat biscuits. For the texture attributes the highest score 8.40 is obtained by potato biscuits followed by wheat and ragi biscuits with the score of 6.90. Regarding the taste attributes the highest score 8.20 is obtained by potato biscuits followed by ragi with a score of 7.60 and the least score is obtained by wheat biscuits is 6.50. For the overall acceptability the highest score 8.40 is obtained by potato biscuits followed by ragi biscuits with a score of 8.00 and least score is obtained by wheat biscuits with a score of 7.20.

Duncan's test reveals that there was a significant difference between the three biscuits for appearance, colour, flavour, texture, taste and overall acceptability at one percent level.

### Nutritive value of the developed biscuits

All the developed biscuits (Table 2) provide more than 500 kilo calories per 100 g of food thus making it calorie rich providing one third of the day's requirement. The protein content of the biscuits is around 7-8 g per 100 g which is also one third of the Recommended Dietary Allowance (RDA) [8] of children in the age group 1-3 years. Therefore, both calories and proteins provided by the biscuits can easily satisfy the day's requirement of children of 2-3 years of age. Presence of good amounts of fat and total ash made the biscuits rich in several macro and micronutrients.

**Table 2:** Nutritive value of the developed biscuits

Nutrients/100 g of food	Potato biscuits	Wheat biscuits	Ragi biscuits	RDA*
Calories (k.cal)	605.89	596.94	546.02	1240

Protein (g)	7.43	8.27	6.03	22

Fat (g)	38.77	37.90	30.20	25

Carbohydrate (g)	56.81	55.69	62.50	-

Crude fiber (g)	2.15	1.88	1.95	-

Total ash (mg)	1.35	1.21	1.22	-

*Recommended daily allowances for 1-3 old children (ICMR 2000)

### Background information of the children

Among the 150 selected children 83 children belonged to the age group of 2 years and 67 children belonged to the age group of 3 years. About the sex 70 were females and 80 were males. Sixty one percent of selected children belonged to nuclear family and rest were in joint families. In majority of the children families (68.1 percent) the family size was 3-4 and as a corollary 56.7 percent of the selected children were of first birth order. All the children selected were from the similar income bracket of about less than 2500 rupees per month.

### Changes in anthropometric parameters before and after biscuits supplementation

#### Improvements in Height

Table 3 gives details regarding the mean increments in height of the children. Highest increment in height (2.16 cm) was recorded by children in Group II given potato biscuits followed by an increment of 0.83 cm recorded by children in Group III given wheat biscuits. The increment observed in the control group (Group I) [9] was only 0.80 cm whereas the increment in Group IV given the ragi biscuits was 0.67 cm. Statistical analysis revealed that these increments between all the groups except control group were significant at five percent level indicating that while wheat and ragi biscuits had an edge over the control in promoting growth, incorporation of potato flour gave further increase in height which showed significantly higher increments with increase in quantity of the food.

**Table 3:** Mean Increments in Height of the selected children

Groups	Height (cms)				
	
	Initial	Final	Difference	Initial Vs Final 't' values	Significance
Group I	73.25 ± 3.35	74.05 ± 4.21	0.80 ± 0.31	0.21	0.59^NS^

Group II	74.21 ± 3.21	76.37 ± 4.21	2.16 ± 1.12	0.03	0.021*

Group III	72.61 ± 4.79	73.44 ± 4.77	0.83 ± 0.44	5.835	0.000*

Group IV	79.31 ± 6.09	79.98 ± 5.90	0.67 ± 0.39	5.409	0.000*

Standard value ** (2-3 years)		81.7			

**NCHS standard for children

*Significant at 5 percent level

^NS^--Not significant

#### Improvements in Weight

The increments in weight (Table 4) in Group II children given potato flour biscuits was higher (1.80 kg) compared to the other three groups followed by Group III with the mean increment of 0.87 kg. Group IV registered a mean increment of 0.55 kg and Group I (control group) registered the least increment of 0.30 kg. When statistically analyzed the difference between the initial and final values of the different groups of children were highly significant (p < 0.05) except control group which showed no significant difference in weight gain.

**Table 4:** Mean Increments in Weight of the selected children

	Weight (kg)				
	
Groups	Initial	Final	Difference	Initial Vs Final 't' value	significance
Group I	11.91 ± 1.28	12.21 ± 1.39	0.30 ± 0.29	0.12	0.056^NS^

Group II	11.61 ± 1.98	13.40 ± 1.89	1.80 ± 0.38	0.07	0.041*

Group III	12.3 ± 1.68	13.17 ± 1.47	0.83 ± 0.36	7.126	0.000*

Group IV	12.14 ± 1.37	12.69 ± 1.38	0.55 ± 0.28	6.128	0.000*

Standard value ** (2-3 years)		10.8			

**NCHS standard for children

*significant at 5 percent level

^NS^- Not significant

### Clinical picture of children

Clinical examinations of the children were carried out initially and finally with the help of the medical practitioner. Initial symptoms like pot belly, oedema, dry skin, dry hair, brittle and spoon shaped nails were prevalent in all the four groups. Supplementation with potato biscuits, wheat and ragi biscuits had wiped out the symptoms of pot belly and oedema (symptoms related to PEM) where as in control group (Group I) there symptoms still existed. Reduction in anaemia related symptoms of brittle and spoon shaped nails were notable in Group II, as against the other groups and this may be a reflection of the increased hemoglobin level exhibited by Group II. In general, reduction in all the clinical symptoms of nutritional disorders was observed in supplemented groups than in the control group.

### Cognitive development of the selected children

Certain developmental characteristics reflecting cognition in 2-3 years old children was studied before and after supplementation period and the data is presented in Table 5. Majority of children (90-100%) in Group II given potato flour biscuits showed good cognition with respect to all the criteria studied, except string beads, folding paper horizontally and building tower after supplementation. A comparatively better picture for cognition was identified in Group III (75-90% children responding) given wheat biscuits as against group IV with 70-85 percent responding and group I with 51-68 percent responding after supplementation. While 48 percent of the children in group II could easily string the beads followed by group III (40%) and group IV (35%) and in Group I they could do so only with great effort (taking more than a minute) after supplementation. While 42 percent of the children in Group II could fold a paper horizontally only 29, 20 and 12 percent of children could do so in group IV, III and I respectively after supplementation. These results are indicative of the development of better cognition in group II children given potato flour biscuits followed by group III children given wheat biscuits than by group IV given ragi biscuits as against the control group again bringing out the importance of potato flour in the total development of children.

**Table 5:** Cognitive Development of the selected children

	Percentage of children							
	
Criteria	Group I		Group II		Group III		Group IV	
	**B**	**A**	**B**	**A**	**B**	**A**	**B**	**A**

1. Knows full name and sex	60	62	59	100	50	89	53	85

2. Uses short sentences (3-4 words)	61	65	62	100	61	85	60	79

3. Recognizes some shapes and colours	59	61	58	100	60	83	62	88

4. Arranges 3 cubes	60	64	62	98	62	80	59	72

5. Follows a short series of direction	50	52	59	92	55	82	52	75

6. Reproduces a circle	50	51	60	90	59	81	58	70

7. Sting heads of large holes with no effort	19	21	20	48	21	40	18	35

8. Understands in, on under	54	58	56	98	55	75	52	72

9. Loves to listen to stories	59	68	48	92	50	88	51	80

10. Develops speech using 'I' and 'Me'	58	60	50	90	51	75	50	72

11. Imitates animal sounds	55	61	51	92	52	90	54	84

12. Identifies objects as same	50	57	55	94	54	88	52	85

13. Folds a paper horizontally	10	12	15	42	11	20	13	29

**B-**Before supplementation

**A**-After supplementation

## Discussion

Studies have documented the positive correlation between the nutritional status of the children with the nutritional knowledge of the parents. As the knowledge is increased better nutritional picture was observed among their children [10-13].

Mean weight of the supplemented children increased by the end of study period compared to the baseline value. Supporting the findings of the present study supplementation of the energy dense foods had good impact on growth status of the children [14]. Similar findings have been reported in children supplemented with the Amylase Rich Flour (ARF) food [15].

In the present study, it was observed that after 3 months of supplementation hemoglobin contents was increased significantly. Owino et al [16] have reported that supplementation of energy dense foods have improved the hemoglobin concentration. In another study supplementation of fortified beverage for 6 months has significantly improved the hematologic and anthropometric measurements and significantly lowered the overall prevalence of anemia deficiency among the children [17]. Similar findings have been reported in various research studies [18].

It is thus evident that in comparison to the control group children the cognitive performance was good in the supplemented group children. This may be due to the fortification of micronutrient dense foods in the weaning biscuits supporting these findings, supplementation of fortified fruit powder beverage for 16 weeks showed significant improvements in cognitive performance [19]. In another study supplementation of beta-carotene fortified biscuits significantly improved the cognitive functions of the children [20].

## Conclusion

In conclusion, all these observations evident that the improvement in anthropometric measurements and better cognitive performance among the malnourished children, clearly indicate that potato flour weaning biscuits undoubtedly helps to improve the nutritional picture and health of the children as that of wheat and ragi biscuits. If such potato flour can form a daily ingredient of the diets of the children right from weaning it can go a long way to help in changing the current malnutrition scenario among the children's and bring out better all round development of the children. Future researches must include development of 100% potato flour, wheat flour and Ragi flour biscuits and its impact should be studied. Alkaloids and cyanogenic compounds should be estimated in the developed biscuits.

## Declaration of Competing interests

The authors declare that they have no competing interests.

## Authors' contributions

PN carried out the design and supervision of the study and manuscript writing and editing. SP performed the field and laboratory studies and statistical data analysis. AH performed clinical support and consultation. All the authors have read and approved the final manuscript.
